# Protective effects of curcumin and niosomal nanocarriers loaded with curcumin on the semen quality, flow cytometry assessments and reproductive performance in aging male broiler breeders during cooling storage

**DOI:** 10.1016/j.psj.2026.107431

**Published:** 2026-07-16

**Authors:** Sarallah Yarmohammadi Barbarestani, Niloofar Nasiri-Foomani, Firooz Samadi

**Affiliations:** Department of Animal and Poultry Physiology, Faculty of Animal Science, Gorgan University of Agricultural Science and Natural Resources, Gorgan, 49138-15739, Iran

**Keywords:** Oxidative stress, Niosomal nanocarriers, Curcumin, Sperm, Fertility

## Abstract

In aging commercial poultry flocks, fertility is often sustained with artificial insemination (**AI**), although cooled semen can result in poor fertility. The objective was to assess effects of curcumin (**Cur**) or niosomal nanocarriers loaded with curcumin (**NNCur**), in concentrations of 100, 200 or 300 μM, on sperm quality and function of cooled rooster semen. hypothesized that supplementing rooster semen extender with NNCur protects sperm from negative impacts of ROS during cooled storage and improves sperm quality and function. Pooled semen samples were diluted in the Beltsville extender, split into seven equal aliquots (Control and three concentrations each of Cur and NNCur), cooled to 5°C and stored for 48 h. Sperm motion, morphology, viability, membrane integrity and functionality, and lipid peroxidation were assessed after 0, 24 and 48 h of cooling. Flow cytometry analyses and AI were conducted using 24 h-cooled semen. Although Cur and NNCur did not affect sperm quality at the onset of cooling storage (0 h), compared to the Control group, extender supplementation with Cur, and in particular NNCur, increased (*P* < 0.05) sperm total and progressive motilities, membrane functionality, viability and mitochondria active potential, and reduced lipid peroxidation at 24 and 48 h. With 24 h cooled-stored sperm, fertility and live sperm were highest (P < 0.05) and late apoptotic and ROS production were lowest (*P* < 0.05) with NNCur (200 and 300 μM). However, hatchability, necrosis, and early apoptosis were not (*P* > 0.05) affected by Cur or NNCur. In conclusion, addition of NNCur to extender mitigated oxidative stress and improved fertility potential of stored chilled rooster semen.

## Introduction

Fertility and reproductive performance of roosters are very important, as only 10% of the broiler breeder flocks are roosters (Behnamifar et al., 2025; Barbarestani et al., 2024b). The breeding period in breeder flocks is normally 64 wk (Maina and Schulze, 2025; Barbarestani et al., 2024a). Broiler roosters begin to ejaculate at ∼25 wk of age, with peak fertility at ∼40 wk. However, declining fertility after 45-50 wk is a problem in broiler breeder flocks (Sarabia Fragoso et al., 2013). A common practice to maintain fertility in aging flocks is to spike (male replacement) breeder flocks at 40 to 50 wk, although this is costly, threatens biosecurity, and disrupts social behavior (Brillard, 2004).

Sperm cooling and freezing are used to preserve rooster sperm for artificial insemination (AI) in commercial flocks. However, AI of hens with cooled semen can result in poor fertility (Sexton, 1977), as the avian sperm plasma membrane is rich in polyunsaturated fatty acids (PUFA), making avian sperm vulnerable to lipid peroxidation (Chen et al., 2024). The addition of antioxidants in sperm extenders reduces lipid peroxidation and prolongs long-term sperm storage (Ros-Santaella and Pintus, 2021; Partyka and Niżański, 2021).

Curcumin, the bioactive compound in turmeric spice, a natural polyphenolic compound with antioxidant properties (anti-inflammatory, antitoxic and anticancer agent), has been used to combat oxidative stress (Motterlini et al., 2000; Jang et al., 2009). For example, curcumin in the diet of aging roosters after peak sperm production decreased sperm MDA concentrations and improved sperm quality and fertility (Kazemizadeh et al., 2019). Similarly, feeding roosters 8 mg/kg turmeric improved sperm motility (Yan et al., 2017). Additionally, curcumin protected cryopreserved human (Santonastaso et al., 2021) and rabbit (Abdelnour et al., 2020) sperm, including diminished intracellular ROS, DNA fragmentation and oxidative stress. However, medical applications of curcumin are limited by its poor solubility, low absorption, and rapid metabolism, resulting in low bioavailability (Grynkiewicz and Ślifrski, 2012).

Currently, there is a growing trend to use nanocarriers, particularly niosomal formulations, to improve bioavailability and solubility of hydrophobic bioactive substances (Shi et al., 2010). In this regard, supplementation of nano-curcumin in diet of aged roosters improved semen quality, fertility, hatchability, and antioxidant status (Behboodi et al., 2024). In addition, curcumin nanoparticles enhanced function of cryopreserved rabbit sperm (Abdelnour et al., 2020). Alpha-lipoic acid-loaded nanostructured lipid carriers improved semen quality and antioxidant capacity, fertility, hatchability of rooster sperm under oxidative stress (Najafi et al., 2020). Recently, some studies demonstrated curcumin-loaded niosomal nanoparticles alleviated oxidative stress in cooled or cryopreserved semen from stallions (Nasiri-Foomani et al., 2024a; Nasiri-Foomani et al., 2024a) and rams (Sadeghi et al., 2024). Similarly, niosomal nanocarriers loaded with artemisinin (a natural antioxidant) enhanced antioxidant capabilities and improved the functional quality of equine sperm by mitigating cryo-oxidative stress (Abbasi et al., 2025). However, there are apparently no reports on using niosomal nanocarriers loaded with curcumin to protect cooled rooster semen.

The objective was to assess effects of Cur or NNCur, in concentrations of 100, 200 or 300 μM, on sperm quality and function of cooled rooster semen. We hypothesized that supplementing rooster semen extender with NNCur protects sperm from negative impacts of ROS during cooled storage and improves sperm quality and function.

## Materials and methods

### Chemicals and ethics

All chemicals used in this study were provided by Merck (Darmstadt, Germany) and Sigma-Aldrich (St. Louis, MO, USA), unless otherwise mentioned. This study was approved by the Animal Care Committee of the Gorgan University of Agricultural Sciences and Natural Resources, Gorgan, Golestan, Iran (Protocol No; 1404/4036019).

### Preparation of NNCur

Preparation of niosomal nanocarriers loaded with curcumin (NNCur) were implemented according to the thin-film hydration technique (Nasiri-Foomani et al., 2024a). Briefly, a mixture of curcumin (0.5 M), Tween 60 (10 mM), cholesterol (2 mM), Span 60 (10 mM) were dissolved in 15 mL chloroform, then agitated overnight at ambient temperature. A rotary evaporator (Heidolph; Germany) was used (60°C and 150 rpm) to evaporate that solution, and the thin-dried film was rehydrated by a phosphate-buffered saline solution (PBS, pH=7.4). To obtain vesicles with uniform distribution, hydrated niosomes underwent sonication (Bandelin, Germany).

### Farm management and semen collection

Semen samples were collected from male broiler breeders, Ross 308, (n = 25; 50 wk of age) housed in individual cages (60 × 50 × 75 cm, length × width × height) under controlled conditions (15 Light: 9 Dark schedule, with 21–23°C). All roosters were fed the same diet that contained 11.5% crude protein, 2,700 kcal ME/kg, 0.7% calcium, and 0.3% available phosphorus, with *ad lib* access to feed and water. Abdominal massage (Burrows and Quinn, 1937) was used for semen collection (twice a week). Semen samples were kept at 37°C and taken to the laboratory. Ejaculates with volume >0.2 mL, concentration >3 × 10^9^ sperm/mL, motility >80%, and >85% morphologically normal sperm were used in this study. Selected samples were pooled and then divided into 7 equal aliquots.

### Semen extender and processing

Beltsville extender (BE), comprised of 0.60 g/L potassium phosphate monobasic (KH₂PO₄), 4.30 g/L sodium acetate trihydrate (C_2_H_9_NaO_5_), 8.61 g/L sodium-l-glutamate (C_5_H_8_NNaO_4_), 0.34 g/L magnesium chloride hexahydrate (MgCl_2_•6H_2_O), 1.95 g/L Tris ((hydroxymethyl) methyl-2- aminoethanesulfonic acid, C_6_H_15_NO_6_S), 0.64 g/L potassium citrate monohydrate (K₃C₆H₅O₇), 12.70 g/L potassium phosphate dibasic trihydrate (K_2_HPO_4_•3H_2_O), and 5.00 g/L d-Fructose (C_6_H_12_O_6_) was selected as the base medium (pH = 7.1 and osmolarity = 310 mOsm/kg) (Sexton., 1977; Nateghi et al., 2024). Semen samples were diluted with extender that was supplemented with Cur or NNCur, according to experimental groups as follows: BE without Cur or NNCur (control), BE with Cur100, Cur200, Cur300, NNCur 100, NNCur 200 and NNCur 300 μM, respectively. Sperm concentration in collected samples was ∼4 × 10^9^ sperm/mL; therefore, semen samples were diluted with the ratio of 1 to 10 in the extender to reach of concentration of 400 × 10^6^ sperm/ml. Semen samples were aspirated into 0.25 mL French straws (IMV, L’Aigle, France), ∼100 × 10^6^ sperm per straw. Subsequently, straws were sealed via polyvinyl alcohol powder, and equilibrated at 5°C up to 48 h. In vitro sperm quality end points were evaluated at 0 (start time), 24 and 48 h after cooling. Flow cytometry analysis, fertility and hatchability were evaluated using 24 -h cooled stored samples.

### Motion parameters

At of 0, 24, and 48 h after storage, sperm motion parameters were evaluated using a sperm class analysis software (Videotest, Sperm 3.2, Russia) modified for avian sperm. Sperm samples were diluted with PBS and incubated at 35°C for 5 min, then 5 mL of diluted semen was placed onto a pre-warmed (37 °C) chamber slide. For each sample, at least six fields that contained a minimum of 400 spermatozoa were evaluated (× 100 magnification). The CASA system was calibrated for: frame rate 60 Hz, number of frames 30, minimum contrast 56, minimum cell size of 5 pixels, VAP cutoff 20.0 μm/s, VAP cutoff for progressive cells 80.0 μm/s, and VSL cutoff 0.0 μm/s. Total motility: fraction of spermatozoa population that displays movement with a straight line velocity >5 μm/s, and progressive motility: fraction of spermatozoa population swimming forward with path velocity >20 μm/s and straightness >80%.The following parameters: total motility (TM, %), progressive motility (PM, %), average path velocity (VAP, μm/s), straight-line velocity (VSL, μm/s), linearity (LIN, %), curvilinear velocity (VCL, μm/s), amplitude of the lateral head displacement (ALH, μm/s) and straightness (STR, %) were recorded after evaluation.

### Plasma membrane functionality (hypo-osmotic swelling test)

To assess sperm plasma membrane integrity, a hypo-osmotic swelling test (HOST) was performed as described (Revell and Mrode, 1994). For this, 20 µl of semen were mixed with 200 µl hypo osmotic solution (4.9 g trisodium citrate, 9.0 g fructose, in 1000 mL of H_2_O with 100 mOsm/kg water). After incubation (30 min), membrane integrity was observed using a phase-contrast microscope (Carl Zeiss, German; 400 × magnification). A total of 200 sperm, with either swollen or non-swollen tails, were identified and categorized as having either intact or compromised membrane integrity, respectively.

### Abnormal morphology

Sperm morphology was assessed after staining with Hancock solution (Schäfer and Holzmann, 2000), comprised of 150 mL of sodium saline (9.01 g of NaCl in 500 mL of distilled water), 62.5 mL formalin (37%), 500 mL double-distilled water and 150 mL buffer solution. First, 50 µl of each sample were added to Eppendorf tubes containing 1 mL of Hancock solution then 10 μL of this mixture was placed on a microscope slide. The rate of sperm with abnormal acrosome (abnormal mid-pieces and tail defects, detached heads, acrosomal and cap abnormalities) was recorded by assessing 200 sperm under a phase-contrast microscope (magnification 1000 ×, oil immersion).

### Lipid peroxidation

The concentration of malondialdehyde (**MDA**) in semen (marker for lipid peroxidation, **LPO**) was assessed using the thiobarbituric acid reaction method (Esterbauer and Cheeseman, 1990). Briefly, 1 mL of diluted sperm was mixed with 1 mL of cold 20% (w/v) trichloroacetic acid to precipitate protein. The precipitation was performed by centrifuging (960 g for 15 min), and 1 mL of the supernatant was incubated with 1 mL of 0.67% (w/v) thiobarbituric acid in a boiling water bath at 100°C for 10 min. After cooling, absorbance was measured at 532 nm using a spectrophotometer (UV-1200, Shimadzu, Japan).

### Flow cytometry analysis

Flow cytometry examinations were evaluated by FACSCalibur (Becton Dickinson, San Khosoz, CA, USA) flow cytometer set (Lee et al., 2008). A minimum of 1 × 10^4^ sperm were examined in each assay at a flow rate of 100 cells/s. The sperm population was obtained through a forward-angle and 90° light scatter to eliminate aggregates and debris. The excitation wavelength was 488 nm, supplied by an argon laser at 250 mW. The fluorescent probes used in the experiment were excited by an argon-ion 488 nm laser. Green fluorescence (Annexin V/Fluorescein Isothiocyanate, **V-FITC**, and Rhodamine 123, **R123**) was detected on the FL1 detector with a 530/30 nm band-pass filter, and red fluorescence propidium iodide (**PI**) was detected on the FL2 detector with a 585/42 nm band-pass filter. In all analyses, sperm were gated based on forward/side scatter signals, and 10,000 cells were acquired per sample. Analysis was performed using FlowJo software (Treestar, Inc., San Carlos, CA).

### ROS production

The method of (Sharma et al., 2017) (Dichlorofluorescin diacetate, DCFH-DA) was used to measure ROS production. The sperm concentration was adjusted to 3-5 × 10^6^ spermatozoa/mL at 25°C (10 g for 8 min). DCFH-DA (25 μM) was added to the sperm suspension and incubated for 40 min at 25°C. Then, suspension was rewashed, and supernatant was removed by diluting the sperm with PBS, then2 μL of PI (PI, 1.0 μg/mL) was added to the semen before analyzing with flow cytometry.

### Apoptosis status

Apoptosis in the sperm after cooling was measured using the Annexin V-FITC kit (IQP, Groningen, Netherlands) according to the company's instructions. Briefly, Sperm samples were washed in calcium buffer and adjusted to a concentration of 1 × 10^6^ spermatozoa/mL. Subsequently, 10 mL of Annexin-V FITC (0.01 mg/mL) was added to 100 mL of the sperm suspension, followed by incubation at 22°C for 20 min. Then, 10 mL PI was added to the sperm suspension, incubated for at least 10 min at 22°C, and finally assessed by a flow cytometer (Ansari et al., 2017). The apoptotic status of the samples was classified as follows: 1) viable cells were negative for both Annexin V and PI (A^-^/PI^-^); 2) early apoptotic cells were positive for Annexin V but negative for PI (A^+^/PI^-^); 3) late apoptotic cells were positive for both Annexin V and PI (A^+^/PI^+^); and 4) necrotic cells were negative for Annexin V but positive for PI (A^-^/PI^+^).

### Mitochondrial activity

The dye of R123 (Invitrogen TM, Eugene, OR, USA) and PI determined mitochondrial activity (Masoudi et al., 2022). In order that a sperm suspension was made using 10 µL of R123 solution, which was added to 300 µL of diluted semen. Then, the suspension was incubated in a dark room for 20 min. Samples were then centrifuged for 3 min at 500 × g and resuspended, again, using 500 µL Tris's buffer. Next, 10 µL of PI (1.0 µg/mL) was supplemented to the sperm suspension. Positive R123 and negative PI samples were recorded as active mitochondria.

### Artificial Insemination

To evaluate fertility and hatchability, artificial insemination (**AI**) was implemented according to the technique of Long and Kulkarni (2004). Seven groups of hens (n = 10 in each group) were kept in individual cages (size= 85 × 70 × 70 cm) and then artificially inseminated with sperm samples containing control, Cur100, Cur200, Cur300, NNCur 100, NNCur 200, and NNCur 300 μM, respectively, at 24 h of incubation. In addition, the data of the beginning time, 0 h, samples were not recorded for AI because the samples had similar quality. Furthermore, the samples that were cooled for 48 h were also not used for AI because of the low quality of 48 h-cooled-stored samples. Straws containing 100 × 10^6^ spermatozoa (1/4 mL) were used for artificial insemination, twice a week for 1 month. We collected the eggs until 5 days after the last insemination. The collected eggs were incubated in the setter for 18 days and transferred to the hatcher for the last 3 days. Fertility rate was measured after 7 days of incubation by candling the eggs [(calculated as the number of fertile eggs divided by the total number of eggs set) × 100]. The hatchability percent of fertile eggs was assessed after 21 days [(calculated as the number of chicks divided by the number of fertile eggs) × 100] (Akhlaghi et al., 2014; Barbarestani et al., 2024c).

### Statistical analysis

Eight semen replicates were used in the current study. The Shapiro-Wilk test was used to assess data normality. Arc sine test was used for the transformation of percentage data when appropriate. Measurement data were analyzed by PROC GLM (SAS, 2002). GENMOD procedure analyzed fertility and hatching rate via Chi-Square. If there were significant (*P* < 0.05) differences between groups were determined using Duncan’s Test.

## Results

### Motility parameters

Effects of semen cooling on sperm motility end points are in Table 1, Table 2, Table 3, Table 4. At time 0 h, there was no difference (*P* > 0.05) among groups for any sperm motility end point. However, after 24 h storage, NNCur200 treatment had highest TM (*P* < 0.01). There were no significant effects among treatments for LIN or ALH. However, after 24 or 48 h, TM, PM, VSL, VCL, and VAP were highest (*P* < 0.01) in all three NNCur groups.

**Table 1:** Effects of different levels of curcumin and NNCur (μM) on rooster sperm total motility (TM, %), and progressive motility (PM, %) during 0, 24 and 48 h of storage at 5°C.

Treatments	Total motility (%)			Progressive motility (%)		
	0 h	24 h	48 h	0 h	24 h	48 h
Control	85.80 ± 1.28	47.60 cd ±0.87	10.20c ±0.37	34.40 ± 0.50	13.80c ±0.96	6.20b ±0.37
Cur100	86.80 ± 0.86	47.00 cd ±1.14	12.80b ±0.86	38.80 ± 0.58	16.40b ±0.24	7.40b ±0.50
Cur200	87.00 ± 0.83	48.20c ±0.66	13.60b ±0.50	38.60 ± 0.40	16.00b ±0.70	7.20b ±0.73
Cur300	88.40 ± 0.87	45.60d ±0.92	13.00b ±1.00	39.00 ± 0.83	16.20b ±0.66	7.00b ±0.31
NNCur100	85.20 ± 1.59	51.60b ±0.67	18.60a ±0.50	39.60 ± 0.50	19.80a ±0.37	9.40a ±0.24
NNCur200	87.20 ± 1.62	54.20a ±0.37	19.60a ±0.92	40.00 ± 0.31	20.80a ±0.37	10.20a ±0.37
NNCur300	88.00 ± 0.77	51.60b ±0.67	19.20a ±0.86	40.20 ± 0.66	21.00a ±0.54	10.00a ±0.54

Abbreviations: Cur, curcumin; NNCur, niosomal nanocarrier loaded with curcumin.

a-d Within columns, different superscript letters indicate significant differences (*P* < 0.05).

**Table 2:** Effects of different levels of curcumin and NNCur (μM) on rooster sperm straight line velocity (VSL, μm/s), and curvilinear velocity (VCL, μm/s) during 24 of storage at 5°C.

Treatments	Straight line velocity (μm/s)			Curvilinear velocity (μm/s)		
	0 h	24 h	48 h	0 h	24 h	48 h
Control	34.20 ± 0.66	24.80c ±0.86	21.40c ±1.07	55.20 ± 0.73	47.20c ±0.80	45.80c ±1.14
Cur100	34.00 ± 0.70	29.40b ±0.74	24.20b ±0.73	54.80 ± 1.35	50.80b ±0.86	47.40bc ±1.28
Cur200	33.80 ± 0.86	29.60b ±1.07	24.60b ±0.92	53.00 ± 1.14	49.80b ±0.66	46.80bc ±1.15
Cur300	32.40 ± 1.02	29.00b ±1.22	24.80b ±0.86	53.20 ± 0.73	50.00b ±0.31	47.20bc ±1.15
NNCur100	32.60 ± 1.20	29.80b ±0.34	30.40a ±0.50	55.80 ± 0.80	56.40a ±0.50	52.40b ±0.74
NNCur200	33.40 ± 1.07	33.60a ±0.50	31.80a ±0.58	54.00 ± 1.51	55.40a ±0.97	54.80a ±0.73
NNCur300	31.60 ± 0.50	32.60a ±0.50	31.40a ±0.87	55.60 ± 0.50	54.80a ±1.01	53.40a ±1.07

Abbreviations: Cur, curcumin; NNCur, niosomal nanocarrier loaded with curcumin.

a-c Within columns, different superscript letters indicate significant differences (*P* < 0.05).

**Table 3:** Effects of different levels of curcumin and NNCur (μM) on rooster sperm average path velocity (VAP, μm/s), and linearity (LIN, %) during 0, 24 and 48 h of storage at 5°C.

Treatments	Average path velocity (μm/s)			Linearity (%)		
	0 h	24 h	48 h	0 h	24 h	48 h
Control	40.80 ± 0.86	30.20c ±0.86	28.80c ±0.37	53.20 ± 1.65	52.80 ± 0.86	47.60 ± 1.28
Cur100	41.00 ± 0.89	33.80b ±1.01	30.80bc ±0.58	53.40 ± 1.43	51.80 ± 0.86	46.40 ± 1.80
Cur200	42.00 ± 0.83	33.40b ±0.74	30.60bc ±0.67	53.80 ± 1.28	53.20 ± 1.15	48.20 ± 1.93
Cur300	41.20 ± 1.24	33.00b ±0.94	32.00b ±0.94	54.00 ± 1.41	52.00 ± 1.22	46.00 ± 1.51
NNCur100	41.80 ± 1.06	37.00a ±0.70	36.60a ±1.02	55.40 ± 2.06	51.60 ± 1.20	44.40±1.02
NNCur200	43.20 ± 0.96	39.00a ±0.70	35.20a ±1.15	54.60 ± 1.02	50.80 ± 0.66	45.80 ± 1.39
NNCur300	43.00 ± 0.89	38.60a ±1.28	37.80a ±0.96	54.59 ± 1.63	50.60 ± 0.74	44.20 ± 0.86

Abbreviations: Cur, curcumin; NNCur, niosomal nanocarrier loaded with curcumin.

a-c Within columns, different superscript letters indicate significant differences (*P* < 0.05).

**Table 4:** Effects of different levels of curcumin and NNCur (μM) on rooster sperm average amplitude of the lateral head displacement (ALH, μm), and straightness (STR, %) during 0, 24 and 48 h of storage at 5°C.

Treatments	Amplitude of the lateral head displacement (μm)			Straightness (%)		
	0 h	24 h	48 h	0 h	24 h	48 h
Control	8.35 ± 0.26	7.33 ± 0.27	6.30 ± 0.26	67.20±0.96	58.20b ±0.86	48.20b ±1.24
Cur100	7.95 ± 0.11	7.57 ± 0.37	6.54 ± 0.37	66.00 ± 2.50	56.80b ±2.20	56.20a ±2.95
Cur200	7.99 ± 0.07	6.89 ± 0.09	5.96 ± 0.26	65.40 ± 2.13	57.00b ±1.76	57.40a ±3.07
Cur300	8.50 ± 0.45	7.50 ± 0.44	6.47 ± 0.42	62.00 ± 1.22	58.20b ±1.74	58.00a ±2.07
NNCur100	8.22 ± 0.33	6.37 ± 0.12	5.87 ± 0.10	63.80 ± 2.35	62.60ab ±1.96	60.60a ±1.86
NNCur200	8.20 ± 0.19	7.21 ± 0.20	6.23 ± 0.18	64.60 ± 1.68	64.80a ±1.68	62.00a ±1.64
NNCur300	7.85 ± 0.20	7.11 ± 0.29	6.12 ± 0.28	61.80 ± 1.49	65.40a ±2.13	63.00a ±2.30

Abbreviations: Cur, curcumin; NNCur, niosomal nanocarrier loaded with curcumin.

a-b Within columns, different superscript letters indicate significant differences (*P* < 0.05).

### Quality parameters and MDA content

Sperm morphological abnormality, viability, membrane integrity functionally and MDA concentrations are in Table 5, Table 6, Table 7. At the start time of experiment, there was no significant difference among groups for membrane functionality, integrity, abnormality or viability.

**Table 5:** Effects of different levels of curcumin and NNCur (μM) on rooster sperm abnormality (%) and viability (%) during 0, 24 and 48 h of storage at 5°C.

Treatments	Abnormality (%)			Viability (%)		
	0 h	24 h	48 h	0 h	24 h	48 h
Control	5.62 ± 0.09	25.68 ± 0.11	44.80 ± 0.08	88.40 ± 0.60	53.40d±0.74	20.80b±0.37
Cur100	5.40±0.13	26.08±0.23	44.74±0.12	89.80±0.96	54.60d±0.24	21.80b±1.06
Cur200	5.50±0.07	25.72±0.10	45.20±0.31	89.00±0.83	55.20cd±0.37	22.40b±1.32
Cur300	5.68±0.08	26.10±0.07	45.22±0.07	90.20±0.37	54.80d±0.58	22.39b±1.36
NNCur100	5.74±0.16	25.90±0.03	44.64±0.16	90.00±0.31	59.00ab±0.54	27.80a±0.37
NNCur200	5.86±0.21	25.80±0.18	45.16±0.30	88.60±0.92	61.00a±0.31	26.50a±0.58
NNCur300	5.72±0.20	25.82±0.05	45.18±0.03	88.00±0.54	57.20bc±1.39	28.20a±0.37

Abbreviations: Cur, curcumin; NNCur, niosomal nanocarrier loaded with curcumin.

a-d Within columns, different superscript letters indicate significant differences (*P* < 0.05).

**Table 6:** Effects of different levels of curcumin and NNCur (μM) on rooster sperm average membrane functionality (MF, %), and membrane integrity (MI, %) during 0, 24 and 48 h of storage at 5°C.

Treatments	Membrane functionality (%)			Membrane integrity (%)		
	0 h	24 h	48 h	0 h	24 h	48 h
Control	78.20 ± 0.37	59.20c ±0.80	15.60c ±0.50	85.00 ± 1.14	48.60c ±0.50	15.80c ±0.48
Cur100	78.60 ± 0.67	58.80c ±0.91	16.20b ±0.58	84.60 ± 0.97	52.00b ±0.83	16.20c ±0.47
Cur200	80.20 ± 0.58	57.60c ±0.24	17.00b ±0.94	84.00 ± 1.14	51.80b ±0.66	15.00c ±0.31
Cur300	79.60 ± 0.92	58.60c ±0.50	16.00b ±0.54	84.20 ± 1.11	51.79b ±0.91	16.40c ±0.24
NNCur100	80.40 ± 0.50	66.20b ±0.20	24.80a ±0.58	85.80 ± 0.96	56.20a ±0.86	22.60b ±0.40
NNCur200	78.80 ± 1.24	65.40b ±0.24	24.79a ±0.37	87.00 ± 0.94	56.80a ±1.06	22.40b ±1.02
NNCur300	79.00 ± 1.26	70.60a ±0.74	26.60a ±0.24	87.00 ± 0.70	58.00a ±1.14	24.20a ±0.20

Abbreviations: Cur, curcumin; NNCur, niosomal nanocarrier loaded with curcumin.

a-c Within columns, different superscript letters indicate significant differences (*P* < 0.05).

**Table 7:** Effects of different levels of curcumin and NNCur (μM) on rooster sperm MDA (nmol/l) during 0, 24 and 48 h of storage at 5°C.

Treatments	MDA (nmol/l(		
	0 h	24 h	48 h
Control	1.48 ± 0.02	3.98a ±0.08	7.12a ±0.08
Cur100	1.51±0.01	3.36bc±0.09	6.08b ±0.15
Cur200	1.52±0.07	3.46b±0.14	6.14b ±0.15
Cur300	1.48±0.01	3.20bc±0.13	6.28b ±0.15
NNCur100	1.47±0.01	3.56b±0.06	4.98c ±0.11
NNCur200	1.52±0.01	3.00c±0.15	4.82c ±0.15
NNCur300	1.50±0.01	3.06c±0.16	5.00c ±0.14

Abbreviations: Cur, curcumin; NNCur, niosomal nanocarrier loaded with curcumin.

a-c Within columns, different superscript letters indicate significant differences (P < 0.05).

Sperm abnormalities were not significantly affected by group at any of the three assessments. However, viability was highest (*P* < 0.05) in NNCur 200 at 24 h at 24 h and was highest in all three NNCur treatments at 48 h.

Membrane functionality was highest (*P* < 0.05) in NNCur 300 at 24 h at 24 h and was highest (*P* < 0.05) in all three NNCur treatments at 48 h. Membrane integrity was highest (*P* < 0.05) in all three NNCur treatments at 24 h and was highest (*P* < 0.05) in NNCur300 at 48 h.

### Apoptosis status, Mitochondrial activity, ROS concentration

**Fig. 1:**
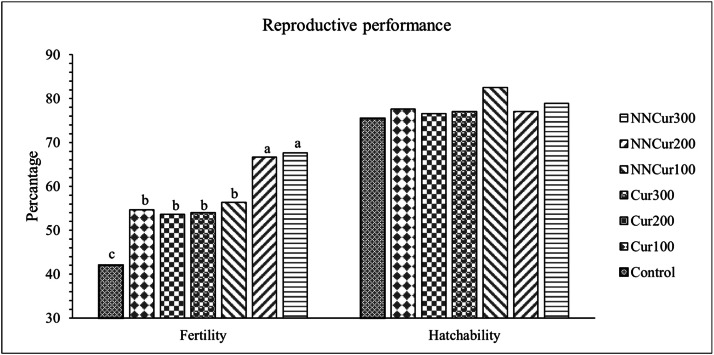
Fertility ((fertile eggs / set eggs) × 100) and hatchability ((hatched eggs / fertile eggs) × 100) rates following artificial insemination (200 × 10^6^ sperm/hen) of hens (10 hens/treatment), by experimental treatments (Control, Cur100, Cur200, Cur300 and NNCur 100, NNCur 200, and NNCur 300 μM) to the diluted rooster semen (during 50-53 weeks of age). Artificial insemination analysis was also conducted using 24 h-cooled semen. Fertility and hatchability rates were analyzed using chi-squared test. Abbreviations: Cur, curcumin; NNCur, niosomal nanocarrier loaded with curcumin. Different letters (a, b) represent significant differences in each parameter (*P* < 0.05).

Fig. 1 presents the results of mitochondrial activity and live, necrosis, early apoptotic, late apoptotic, and ROS concentration at 24 h of storage. Mitochondrial activity rate was higher (*P* < 0.05) in NNCur300 (63.00 ± 0.70%), NNCur200 (59.40 ± 0.92%) than Cur100 (53.00 ± 1.30%), Cur200 (52.80 ± 1.01%), Cur300 (52.2 ± 0.86%) and NNCur100 (59.2 ± 0.86%), finally we observed lowest Mitochondria activity in control group (51.2 ± 0.66%).

The percentage of live sperm was significantly higher in NNCur200 and NNCur300 compared to other groups (*P* < 0.05). Also, the control group produced the highest significant percentage of late apoptosis sperm compared to other groups (*P* < 0.05). Likewise, the results showed no significant differences among different groups for the percentage of early apoptosis sperm and necrosis (*P* > 0.05).

NNCur100, NNCur200, and NNCur300 significantly had less amount in ROS production than other groups (*P* < 0.05).

### Fertility and hatchability rate

**Fig. 2:**
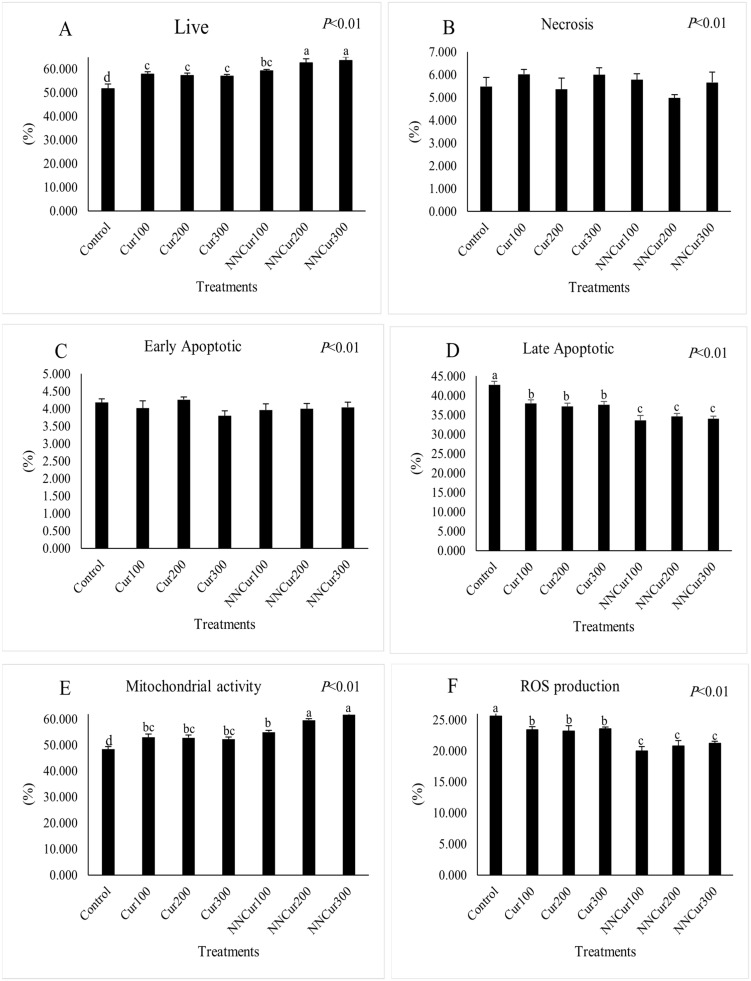
The percentages (LSmean ±SEM) of rooster sperm Live (A), Necrosis (B), Early Apoptotic (C), Late Apoptotic (D), Mitochondrial function (E) and ROS production (F) by experimental treatments (Control, Cur100, Cur200, Cur300 and NNCur 100, NNCur 200, and NNCur 300 μM) during 24 h of storage at 5°C. Different letters (a, b, c) in the bars indicate significant differences. (*P* < 0.05). Error bar = standard error of the mean.

Fig. 2 presents the effects of experimental treatments on the fertility and hatchability rate of rooster sperm at 24 h storage at 5°C. Although the effect of different levels of Cur and NNCur on hatchability rate was not significant. A significantly higher (*P* < 0.05) percentage of fertility rates was observed in NNCur200 and 300 compared to the other groups (62.00%, 62.50% and 63.9% vs. 50.3%, 47.5%, 49.7%, 51.1%, Fig. 1), on the other hand, we observed the lowest fertility in the control group.

## Discussion

Semen cooling or freezing and AI are used to improve reproductive performance in aging commercial flocks or preserve genetic resources (Shahverdi et al., 2015; Sharideh et al., 2019; Chen et al., 2024 Masoudi et al., 2025). Rooster sperm is more sensitive to oxidative stress compared to other species (Fattah et al., 2018; Safari Asl et al., 2018) as the membrane of poultry sperm is sensitive to cold shock due to a high concentration of phospholipids (Sun et al., 2021; Parks and Lynch, 1992). In an attempt to alleviate cooling-induced oxidative stress on semen quality, and thereby to increase fertility and improve reproductive performance, antioxidants as nanocarriers (high bioavailability) can be added to semen. In the present study, Beltsville extender with Cur and specifically NNCur (200 and 300 μM) produced the highest motility, viability, mitochondria active, membrane functionality and integrity as well as the lowest lipid peroxidation, late apoptotic, ROS production. Furthermore, after AI of hens with 24-h cooled stored semen, fertility rate was highest in NNCur100 and NNCur200. This enhancement was attributed to the enhanced relative bioavailability of the niosomal nanocarriers containing curcumin.

Curcumin is a natural antioxidant, reducing oxidative stress, and having anti-inflammatory, antitoxic and anticancer properties (Motterlini et al., 2000; Jang et al., 2009). Curcumin reduced oxidative stress in cooled or cryopreserved semen of goats (Ismail et al., 2020), rams (Ömür et al., 2014), rats (Abdelnour et al., 2020), and boars (Chanapiwat and Kaeoket et al., 2015). However, curcumin has many limitations and drawbacks, including low bioavailability, and solubility which might be due to low water solubility, poor absorption, and rapid metabolism and elimination (Sharifi-Rad et al., 2020; Ahmed-Farid et al., 2017). Currently, there is a growing trend to use nanocarriers, particularly niosomal formulations, to improve bioavailability and solubility of hydrophobic bioactive substances (Shi et al., 2010). Antioxidant-loaded niosomal nanoparticles reduced oxidative stress in cooled and cryopreserved equine (Nasiri-Foomani et al., 2024ab, Abbasi et al., 2025) and ovine (Sadeghi et al., 2024) semen.

Sperm motion end points (TM, PM, VAP, VSL, VCL, ALH, and STR) are influenced by the ability of mitochondria to generate ATP and the functionality of the plasma membrane (Ghadimi et al., 2014); both of these factors were highest in sperm treated with NNCur.

The improved sperm motility parameters observed after 24 and 48 hours of cooled storage may be due to the enhanced bioavailability of nano-curcumin. Smaller particle size increases wettability by expanding the specific surface area, which significantly boosts the solubility and dissolution rate of NNCur. Nano-sized particles present a larger surface area, facilitating greater exposure of hydroxyl groups on their molecular surface. Consequently, individual molecules are more effectively solubilized through solvation, forming hydrogen bonds with water molecules. This process enhances their solubility and bioavailability (Kanwal et al., 2023; Hettiarachchi et al., 2021).

Similarly, curcumin nanoparticles reduced oxidative stress, improving mitochondrial activity and the antioxidant enzyme system (Sadeghi et al., 2024; Nasiri-Foomani et al., 2024a, 2024b; Ismail et al., 2020; Abdelnour et al., 2020). In this study, membrane functionality (HOST) and acrosome integrity were highest with Cur-NN200 and Cur-NN300, similar to previous reports (Sadeghi et al., 2024; Behboodi et al., 2024). Increased acrosome integrity and functionality of sperm could result in better characteristics of sperm such as motility.

Sperm morphological abnormalities were not significantly different among groups. It has been reported that in vitro manipulation has no impact on sperm morphology as this is largely determined during spermatogenesis (Chenoweth, 2005). In this trial, reduced MDA concentrations protected sperm from membrane damage by inhibiting lipid peroxidation. Similarly, several studies reported that adding Cur or NNCur to extender reduced lipid peroxidation in cryopreserved sperm (Sadeghi et al., 2024; Khalil et al., 2025; Abdelnour et al., 2020; Nasiri-Foomani et al., 2024a, 2024b).

Based on flow cytometry, Cur and particularly NNCur provided protective benefits against cryo-induced mitochondrial damage by attenuation of ROS production. After 24 h of cooling, viability and apoptosis status indicated that the addition of NNCur to the semen extender increased the number of living sperm, manifested by a reduction in the proportion of late-apoptotic sperm cells, as well as reduced ROS production in NNCur treatments. This protective effect seemed to be associated with decreased lipid peroxidation of the plasma membrane. In the present study, increased viability was attributed to an antiapoptotic effect, which increased sperm motility and velocity. Similarly, earlier studies highlighted beneficial effects of nano-curcumin on apoptosis status and viability of oxidative stress in sperm from rams (Sadeghi et al., 2024), stallions (Nasiri-Foomani et al., 2024a, 2024b), goats (Ismail et al., 2020) and rabbits (Abdelnour et al., 2020). These enhancements were attributed to the increased relative bioavailability and the subsequent improvement in the antioxidative properties of the niosomal nanocarriers that incorporate curcumin. The reduction in both MDA and ROS concentrations, coupled with increased sperm viability, as observed in the current study, further supported this assertion.

Fertility evaluation is very important to confirm *in vitro* results (Graham et al., 2005). The sperm storage tubules (SSTs) are located in the utero-vaginal junction of the hen’s oviduct where inseminated sperm are gradually released. Sperm quality is very important, as only high-quality sperm can reach the SST. However, cooling poultry sperm can induce DNA fragmentation and trigger apoptosis due to oxidative stress (ROS), thereby reducing fertility (Amini et al., 2015). Exogenous antioxidants added to extender solution may sustain sperm viability and motility, facilitate sperm movement through the oviduct. Furthermore, protection against oxidative stress could improve sperm integrity during sperm transportation (Askarianzadeh et al., 2018). In the current study, the highest fertility rate was 66.7 and 67.7% when hens were inseminated with 24 h-cooled-stored semen samples that contained 200 and 300 µM NNCur, respectively; these high fertility rates were attributed to good semen quality end points. However, hatchability rate was not significantly influenced by Cur or NNCur. Similarly, in previous studies, hatchability was not affected by sperm quality (Feyzi et al., 2018; Sharideh et al., 2019; Masoudi et al., 2025). Various external factors can affect hatching rates in birds, including temperature and humidity of incubator, egg management on farm, egg transportation and eggshells quality.

## Conclusions

In this study, Cur and in particular NNCur, improved motility, sperm plasma membrane functionality and integrity via mitochondria activity preservation and lipid peroxidation reduction. Additionally, there were lower percentages of necrotic cells, as well as lower concentrations of MDA and ROS, compared to the control group, which in turn enhanced the fertility rate of rooster cooled-stored semen. Therefore, Cur, and in particular NNCur, might be considered as a novel potential antioxidant protective for the storage of rooster sperm in cooling condition. The best improvement in sperm quality and fertility rate were with NNCur 200 and 300 μM. Therefore, this approach has potential to sustain quality of cooled rooster semen for AI, as a means to sustains fertility and reproductive performance without male replacement, in aging commercial flocks, thereby improving biosecurity and animal welfare and increasing profitability and sustainability.

## Disclosures

The authors have no conflicts of interest to disclose.
